# Identify and validate a novel ubiquitination-related biomarker for thyroid cancer prognosis and immunotherapy

**DOI:** 10.3389/fonc.2025.1542784

**Published:** 2026-01-16

**Authors:** Yilin Li, Jutao Zeng, Xiongmeiyu Gong, Wei Li, Jueli Liu, Dan Li, Yangyan Zhong, Junjie Deng, Jian Deng, Haigang Feng, Jie Luo, Hong Cao

**Affiliations:** 1 Department of Breast and Thyroid Surgery, The Second Affiliated Hospital, Hengyang Medical School, University of South China, Hengyang, Hunan, China; 2 Department of Thyroid, Breast, Hernia and Anorectal Surgery, Beijing Fengtai Hospital, Beijing, China; 3 School of Medicine, Hunan Normal University, Changsha, Hunan, China; 4 Department of Anesthesiology, The Second Affiliated Hospital, Hengyang Medical School, University of South China, Hengyang, Hunan, China

**Keywords:** thyroid cancer, ubiquitination, tumor microenvironment, immunotherapy, prognosis

## Abstract

**Background:**

Ubiquitination is a critical protein modification process that plays a pivotal role in maintaining cellular homeostasis and is implicated in various pathophysiological processes, including thyroid cancer (THCA). Understanding the roles of ubiquitination-related genes in THCA progression and their interactions with the tumor microenvironment (TME) could provide valuable insights into prognosis and treatment strategies.

**Methods:**

Using iUUCD 2.0, ubiquitination-related genes were identified and subjected to consensus clustering on TCGA-THCA data. Differentially expressed genes (DEGs) between tumor and normal tissues were identified and used to construct a ubiquitination-related signature using Cox and LASSO regression. The signature’s prognostic ability was validated using training and test datasets from TCGA. Immune cell infiltration, immunotherapy response, and drug sensitivity were analyzed.

**Results:**

Three ubiquitination clusters were identified among 454 genes. Four prognostic DEGs (F12, FBXO15, FBXW10, and USP44) formed the signature, significantly correlating with survival, immune cell infiltration, clinical characteristics, immune checkpoints, immunotherapy response, and drug sensitivity. Low-risk THCA patients had better prognosis and immunotherapy responses than high-risk patients. A stable nomogram combining the signature and clinical characteristics predicted patient survival. RT-qPCR and immunohistochemistry confirmed differential expression of key genes.

**Conclusion:**

Our study identifies and validates a novel four-gene ubiquitination-related signature as a promising and independent prognostic biomarker in THCA. Beyond outcome prediction, this signature demonstrates significant translational potential by accurately predicting immunotherapy responses, thereby facilitating the development of more personalized and effective treatment strategies for patients with THCA.

## Introduction

Thyroid cancer (THCA) is the most prevalent malignancy of the endocrine system, with papillary thyroid carcinoma (PTC) being the dominant subtype, followed by follicular, medullary, and anaplastic thyroid carcinomas (1, 2). The global incidence of THCA has steadily increased, especially in women, with recent epidemiological data showing rates of 22.0 and 7.4 per 100,000 in females and males, respectively, in the United States (3, 4). Although most cases of THCA exhibit indolent behavior and favorable outcomes, aggressive subtypes such as anaplastic and medullary thyroid carcinomas, as well as radioiodine-refractory THCA, are associated with recurrence, metastasis, and poor prognosis (5). In clinical practice, targeted therapies such as sorafenib—an inhibitor of VEGFR, BRAF, and RET—are used to treat advanced or metastatic THCA. However, their therapeutic benefit remains limited by toxicity and suboptimal response rates (6).

Recent advances in immunotherapy have revolutionized cancer treatment paradigms; however, only a subset of patients with THCA exhibit durable responses (7, 8). This hindrance underscores the urgent need to identify robust biomarkers that can stratify patients by prognosis and therapeutic responsiveness. Moreover, emerging models integrating imaging features and locational data have improved preoperative risk prediction, such as for central lymph node metastasis (CLNM), thereby further underscoring the value of precision medicine approaches in THCA (9). Therefore, novel and reliable genes should be identified to assess tumor aggressiveness and biological behavior, and to provide important guidance for the precise treatment of patients with THCA and poor prognosis.

Post-translational modification (PTM) is a covalent process in which proteins are modified by adding chemical groups and altering hydrolysis time to remove modifying groups, thereby affecting their properties (10). The main forms of PTM include phosphorylation, glycosylation, acetylation, ubiquitination, carboxylation, ribosylation, and disulfide bond pairing (11–13). Ubiquitination is a widespread PTM mode that is highly correlated with autophagy (14, 15). The E1 enzyme is responsible for the adenylation of ubiquitin, a process that consumes one molecule of ATP, and the E2 enzyme transports ubiquitin that is adenylated by E1 to E3 (16). Different E3 enzymes recognize diverse substrate proteins and catalyze ubiquitin transfer from E2 to the lysine (K) residues of substrate proteins to complete the monoubiquitination or polyubiquitination of the proteins (17, 18).

Ubiquitination regulates numerous oncogenic processes, including cell cycle progression, apoptosis, and immune evasion, pathways that critically determine tumor behavior and treatment response (19). For example, ubiquitin-conjugating enzymes (E2s) such as UBE2C regulate normal cell proliferation; however, their overexpression, frequently observed in thyroid and other cancers, has been associated with aberrant cell cycle progression, enhanced tumor invasiveness, and metastasis (20–22). Moreover, components of the ubiquitination machinery, such as E3 ligases and DUBs, are druggable targets, and inhibitors of specific E3s or deubiquitinating enzymes (DUBs) have already shown therapeutic promise in preclinical models of other cancers (23). Regarding THCA, the deltex E3 ubiquitin ligase 4 (DTX4) has been reported to promote cancer progression by regulating stearoyl-CoA desaturase 1 (24–28). RNF115 is upregulated in papillary thyroid carcinoma (PTC) and promotes cell proliferation and invasion by ubiquitinating and degrading cyclin-dependent kinase 10 (CDK10), thereby activating the Raf-1 pathway (29). Conversely, RNF150 is downregulated in PTC and exerts a suppressive role by targeting apoptosis signal-regulating kinase 1 (ASK1) for ubiquitination and degradation, thereby reducing p38 phosphorylation and inhibiting cell proliferation and migration (30). Given this dual role in cancer progression and therapeutic vulnerability, ubiquitination-related genes are attractive candidates for prognostic modeling and therapeutic guidance in THCA. Despite increasing evidence linking individual ubiquitination regulators to tumorigenesis, the prognostic and therapeutic relevance of ubiquitination-related genes as a group remains underexplored in THCA.

In this study, transcriptomic data from TCGA and GEO were integrated with a curated list of E3 ligases and DUBs from the iUUCD 2.0 database to identify ubiquitination-related genes associated with THCA prognosis. Through consensus clustering, molecular subtypes with distinct immune landscapes and survival outcomes were identified. Subsequently, a four-gene ubiquitination-related signature was constructed and validated for risk stratification and immunotherapy responsiveness prediction. Functional enrichment and drug sensitivity analyses further elucidated potential mechanisms and therapeutic implications. These findings provide novel insights into the role of ubiquitination in THCA progression and support the utility of ubiquitination-related signatures in guiding personalized treatment strategies.

## Materials and methods

### Data collection and processing

THCA mRNA expression profiles and corresponding clinical data, including 510 tumor samples and 56 normal tissue samples, were obtained from the UCSC Xena browser (https://xenabrowser.net/datapages/). The fragments per kilobase of exon model per million mapped fragments (FPKM) values were normalized with the transcripts per million (TPM) method and converted (log2 + 1) in TCGA. The GSE33630 dataset (60 cancers vs. 45 normal) and GSE60542 (58 cancers vs. 34 normal) cohorts were downloaded from the GEO database (http://www.ncbi.nlm.nih.gov/geo) (31).

A total of 576 E3 genes and 95 DUB genes were identified in the iUUCD 2.0 (http://iuucd.biocuckoo.org/) database (**Supplementary Table S1**) (32). The TCGA genes were intersected with these genes, resulting in 454 genes for subsequent analysis. TCGA-THCA was randomly divided into a training group and a test group at a 1:1 ratio by using R software. Each set has 255 samples.

### Identification of ubiquitination clustering

Consensus clustering was performed to identify optimal ubiquitination clusters and to reveal the expression patterns of ubiquitination-related genes in THCA. The expression of 454 ubiquitination genes was used for consensus clustering with the “ConsensusClusterPlus” R package (k=3) (33). A consensus matrix and a cumulative distribution function (CDF) were used to determine the optimal number of clusters. The Kaplan–Meier (K-M) method was performed between the subtypes to determine the survival differences between clusters. The ‘ssGSEA’ package was used to calculate the immune infiltrating cell score for the TCGA cohort. The score was used to compare immune function and immunological pathways between the clusters. Different immune cell types, such as natural killer cells, activated CD4 T cells, CD56 bright natural killer cells, type 2 T helper cells, CD56 dim natural killer cells, plasmacytoid dendritic cells, central memory CD4 T cells, gamma delta T cells, type 1 T helper cells, central memory CD8 T cells and effector memory CD8 T cells were also analyzed.

### Identification and confirmation of ubiquitination-related genes in THCA

Differentially expressed genes (DEGs) in tumor tissues and adjacent normal tissues in the TCGA THCA cohort were analyzed by the DESeq2, edge, and limma packages in R (version 4.3.2; https://cran.r-project.org/). Genes with (|log2FC|> 2 and p < 0.05 were considered significantly different. The R Venn package was used to identify crossover genes among DEGs, yielding ubiquitination-related DEGs in THCA (**Supplementary Table S2**).

### Establishment of an ubiquitination-related signature

Univariate Cox regression analysis was performed to identify prognostic DEGs, with HR < 1 or > 1 indicating protective or risk genes (p < 0.05). Least absolute shrinkage and selection operator (LASSO) regression analysis was performed to identify signature genes by using the ‘glmnet’ package to avoid overfitting (34). The risk score was calculated using the following formula: ∑nnCoef(i)*Expr(i).

Patients with THCA were divided into low- and high-risk subgroups based on their risk score relative to the median risk score. K-M survival analysis was conducted to evaluate the prognosis of patients with low- and high-risk THCA. A receiver operating characteristic (ROC) curve was used to confirm prediction stability (35). Clinical traits were further combined with univariate and multivariate Cox regression analyses by using the ‘survival’ package. The variables that were analyzed were age, gender, stage, T stage, N stage, and risk score. Cox regression was used to analyze the relationship between each clinical trait and survival probability. The Wilcoxon test was used to calculate the relationship between each clinical trait and risk scores.

### Nomogram construction

A nomogram was constructed based on age, sex, T stage, N stage, clinical stage, and ubiquitination-related signature to improve the clinical value (36). A calibration curve was constructed to show the relationship between the actual and predicted probabilities for 1-, 3-, and 5-year OS. The discriminative ability and prediction accuracy of the nomogram were evaluated by the consistency index (C-index). The C-index was calculated by 1000 bootstrap resamples. The values are typically stratified into four tiers: <0.5 (low accuracy), 0.5–0.7 (moderate accuracy), 0.7–0.9 (high accuracy), and >0.9 (extreme accuracy). The clinical usefulness of the nomogram was evaluated using the Decision Curve Analysis (DCA) curve. The discrimination performance of each factor for THCA was evaluated using ROC analysis.

### Clinical correlation analysis

Subgroup analyses of the training dataset were conducted, including age, sex, T stage, M stage, N stage, and clinical stage, to explore the correlation between the related signature and several clinical characteristics. Survival difference between low- and high-risk THCA in distinct clinical subgroups was evaluated using K-M survival analysis. Moreover, the generalizability of the TCGA-derived signature was tested on the independent GSE165724 and GSE150899 datasets. GSE165724 contained expression profiles of 16 PTC, 46 tumor-adjacent thyroid tissues, and 12 normal thyroid tissue samples. GSE150899 contained expression profiles of 10 paired thyroid cancer tissues, including normal tissues, tumor tissues, and metastatic lymph nodes. Risk scores were calculated for each sample and were compared across different tissue types.

### Immune cell infiltration and immunotherapy response

For immune analysis, ssGSEA and CIBERSORT were used to evaluate the extent of immune cell infiltration in different samples. For ssGSEA, the source of the immune gene set was obtained from Charoentong et al.’s research (37) which contained 24 genes (PDCD1, CD274, PDCD1LG2, CTLA4, CD80, CD86, LAG3, HAVCR2, TIGIT, BTLA, CD160, TNFRSF4, TNFRSF9, TNFRSF18, ICOS, ICOSLG, CD40, CD40LG, CD70, CD27, CD276, VTCN1, HHLA2, and SIGLEC15). The original TCGA-THCA data were converted to Log2(CPM + 1) (38). For CIBERSORT, the number of iterations was set at 1000. Samples with calculation results of P value < 0.05 were removed. The ESTIMATE algorithm (39) was used to calculate the immune, stromal, and ESTIMATE scores to reflect the TME status. Immune cell infiltration was assessed using the CIBERSORT algorithm with different colors to explore the relationship between immune cell infiltration and ubiquitination-related signatures (40). The original TCGA-THCA data were converted to Log2(TPM + 1). The expression of immune checkpoints in low- and high-risk THCA was analyzed using the limma algorithm (|log2FC|> 2, p < 0.05). Immune cells, including CD8 T cells, cytotoxic lymphocytes, endothelial cells, fibroblasts, monocytic lineage, myeloid dendritic cells, neutrophils, T cells, and NK cells, were also analyzed in low- and high-risk THCA and presented in bar plots.

Tumor immune dysfunction and exclusion (TIDE), myeloid-derived suppressor cells, and immunophenoscore (IPS) were calculated to predict immunotherapy response in the two subsets.

### Functional enrichment analysis

Gene Ontology (GO) and Kyoto Encyclopedia of Genes and Genomes (KEGG) analyses were conducted on DEGs in low- and high-risk THCA, identified using the limma algorithm (|log2FC| > 2, p < 0.05), to explore underlying putative mechanisms (41, 42). Gene set enrichment analysis (GSEA) was performed to assess differences in pathway activity between low- and high-risk THCA (p < 0.05) (43). The annotated file ‘c6.all.v2023.2.Hs.symbols.gmt’ was downloaded from MSigDB. Functional enrichment analyses were conducted using the ‘ClusterProfiler’ R package (p <0.05) (44).

### OncoPredict for drug sensitivity analysis

The OncoPredict R package was developed by Maeser et al. (45) to predict *in vivo* drug responses in cancer patients. This package fits the gene expression profile of tissues to the half-maximal inhibitory concentration (IC50) of drugs for cancer cell lines from the Genomics of Drug Sensitivity in Cancer (GDSC; https://www.cancerrxgene.org/) and to the gene expression profile of cancer lines from the Broad Institute Cancer Cell Line Encyclopedia (CCLE; https://portals.broadinstitute.org/ccle_legacy/home). A total of 198 drugs were calculated, and sensitivity (between the high- and low-risk groups) was analyzed using unpaired t-tests. p < 0.05 was set as the threshold for significance.

### Exploration of signature genes in databases

The limma algorithm (|log2FC| > 2, p < 0.05) was used to calculate mRNA differences between normal and tumor samples to further explore the expression of the four signature genes in THCA and GEO. The TCGA-THCA, GSE60542, and GSE33630 datasets were extracted for analysis (46).

### Patient samples

All samples (THCA and adjacent normal tissues) were obtained from Nanhua University Second Hospital (Henyang, Hunan, China) between November 2023 and December 2023 (*n* = 10). All pathological features were confirmed by experienced pathologists, and none of the patients received preoperative anticancer treatment.

### RNA extraction and quantitative real-time polymerase chain reaction

TRIzol reagent (Invitrogen) was used to extract total RNA. Reverse transcription was performed using Random Primer and M-MLV Reverse Transcriptase (Promega, Madison, WI, USA) as directed by the manufacturer. Real-time quantitative PCR (RT-qPCR) was performed using SYBR Green PCR Master Mix (Applied Biosystems; Thermo Fisher Scientific, MA, USA) on the CFX384 Real-Time System (Bio-Rad, CA, USA). All the primers are listed in **Supplementary Table S3**. GAPDH was used as an endogenous control.

### Immunohistochemistry

Immunohistochemistry (IHC) was performed as previously described (47). F12 antibody (A1691, abclonal), FBXW10 antibody (bs-16054R, Bioss), USP44 antibody (A15528, abclonal) and FBX15 antibody (bs-8396R, Bioss) were used at 1:150 dilutions. The secondary antibody was a peroxidase-labelled antibody (rabbit IgG (H+L) KPL, Baltimore, MD, USA). Finally, the sections were incubated with DAB solution for 10 min at room temperature. The average optical density of each sample was determined by ImageJ software (NIH, MD, USA).

### Statistical analysis

The analysis and relevant figures were obtained using R software (version 4.3.2). A t-test was used to evaluate the differences between the two groups. Spearman’s analysis was used to calculate correlation coefficients. Kaplan–Meier survival analyses with log-rank tests were performed to assess significant differences in OS between the two groups. The statistical significance was set at p < 0.05.

## Results

### Identification of three ubiquitination clusters and Evaluation of immune cell infiltration

**Figure 1:**
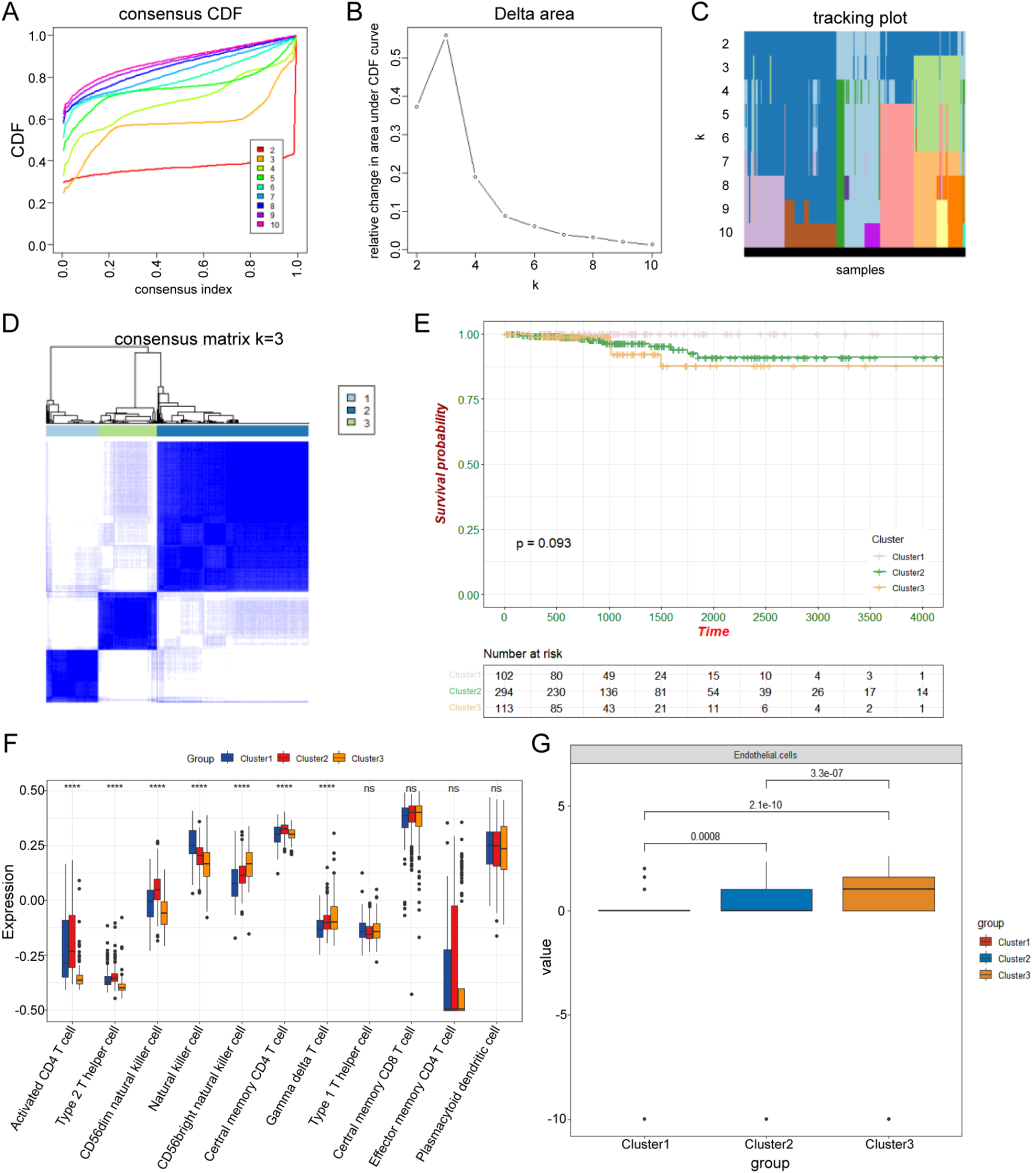
Identification of the three ubiquitination clusters. **(A)** Consensus CDF in consistent clustering (k = 2–10). **(B)** Relative change in area under the CDF curve from k 2–10. **(C)** Tracking plot of the THCA samples (K = 2–10). **(D)** Consensus heatmap defining the three clusters (k = 3). **(E)** K-M survival analysis showing a significant prognosis between the three clusters. **(F, G)** The ssGSEA and MCPCOUNTER algorithm was used to explore immune cell infiltration in the three clusters. *** p<0.005; ns, not significant.

Consensus clustering results showed a significant difference when k = 3, and the curve has a gentle slope (**Figures 1A–C**). Therefore, patients in TCGA-THCA were divided into clusters 1, 2, and 3. The heatmap shows that the three clusters have clear edges (**Figure 1D**). K-M survival analysis was performed among the three clusters to determine whether different expression patterns of ubiquitination-related genes affect the prognosis of THCA. The results showed that cluster 1 had the best outcome, and cluster 3 had the worst (**Figure 1E**). The ssGSEA and MCPCOUNTER algorithm were used to explore immune cell infiltration in the three clusters (**Figures 1F, G**). ssGSEA analysis showed Cluster 1 contained the highest number of natural killer cells. Cluster 2 showed the highest numbers of activated CD4 T cells, CD56 dim natural killer cells, type 2 T helper cells, and central memory CD4 T cells. Cluster 3 had the highest number of CD56 bright natural killer cells, gamma delta T cells (**Figure 1F**). The MCPCOUNTER analysis showed that cluster 1 has the lowest endothelial cell infiltration, suggesting the lower angiogenesis (**Figure 1G**). The high proportion of NK cells and low proportion of endothelial cells may partly account for a favorable prognosis.

### Identification of ubiquitination-related DEGs in the TCGA cohort

**Figure 2:**
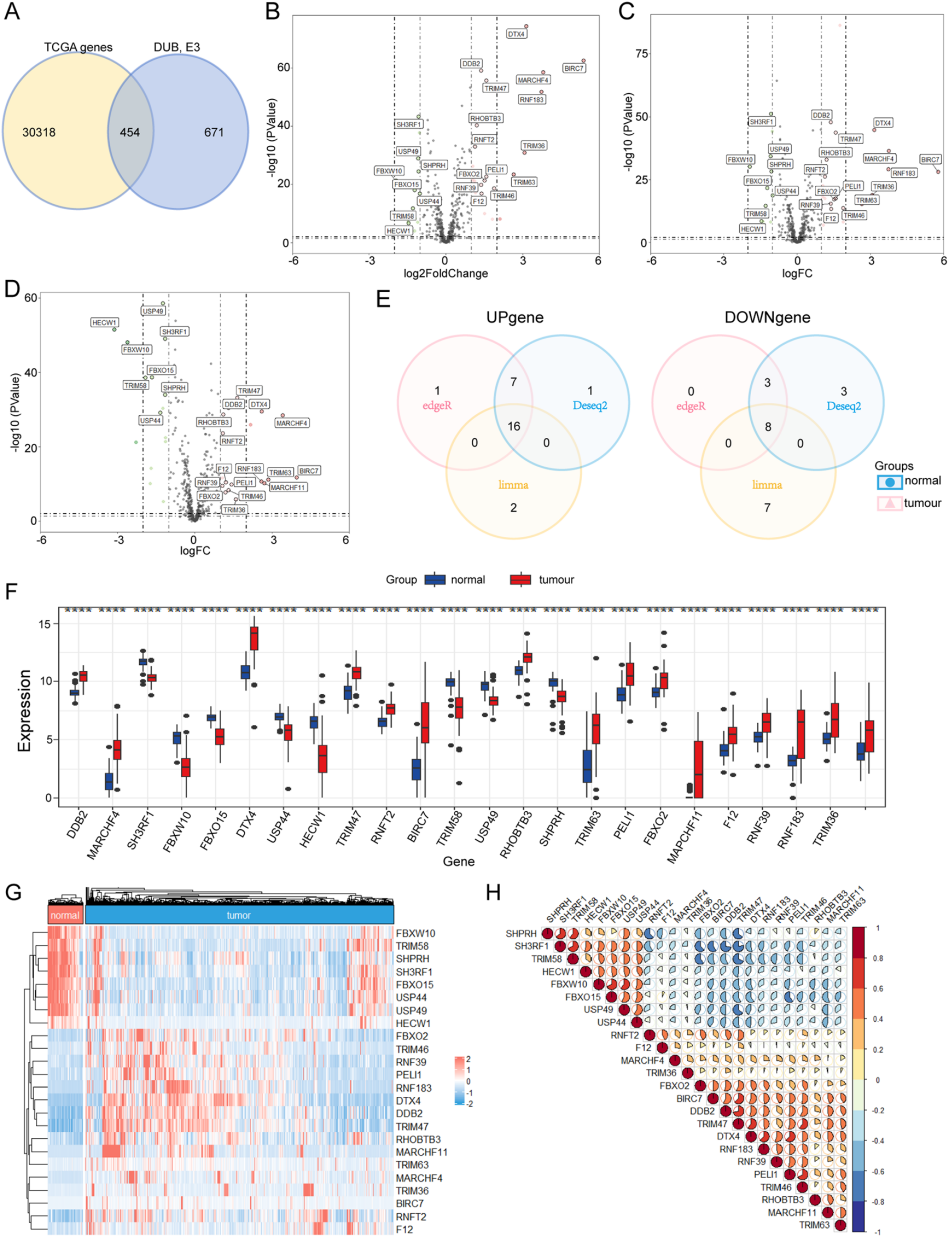
Identification of candidate ubiquitination-related genes in the Cancer Genome Atlas (TCGA) cohort. **(A)** Venn diagrams to identify differentially expressed ubiquitination-related genes between the TCGA cohort and DUB, E3. **(B–D)** Differentially expressed genes between THCA and adjacent normal thyroid tissue are shown by a volcano plot in different ways. **(E)** Venn diagrams to identify up genes and down genes in three different ways. **(F)** Boxplot showing the differentially expressed ubiquitination-related genes in tumour and normal tissues. **(G)** Heatmap showing the differentially expressed ubiquitination-related genes in tumour and normal tissues. **(H)** The correlation between 24 ubiquitination-related genes and 24 ubiquitination-related genes. **** p<0.001.

454 ubiquitination-related genes were included by intersecting 576 E3 genes and 95 DUB genes, and 24 DEGs were obtained by taking the intersection of the results of three different methods, indicating that 24 ubiquitination-related DEGs were differentially expressed in thyroid cancer tumor tissue and adjacent normal tissue (|Log2FC|>2, p<0.05, **Figures 2A–D**). In the TCGA THCA cohort, 24 DEGs were identified in tumor and adjacent normal tissues, of which 16 were upregulated in tumor tissues and 8 were downregulated (**Figure 2E**). As shown in the bar diagram and heatmap, 24 genes were significantly different between normal and tumor tissues. TRIM47, DDB2, DTX4, MARCHF4, RHOBTB3, RNFT2, BIRC7, TRIM63, RNF183, F12, MARCHF11, PELI1, RNF39, TRIM46, FBXO2 and TRIM36 were significantly upregulated, whereas USP49, HECW1, SH3RF1, FBXW10, FBXO15, TRIM58, SHPRH and USP44 in the TCGA-THCA cohort (**Figures 2F, G**) were downregulated. The Spearman correlation analysis supported the relationship between ubiquitination-related genes (P<0.05), such as FBXW10 and FBXO15, FBXW10, USP49, TRIM47, and DTX4, which were significantly positively correlated; TRIM47 was negatively correlated with USP49, and SHPRH was negatively correlated with RNFT2 (**Figure 2H**).

### Identification of ubiquitination-related signature

**Figure 3:**
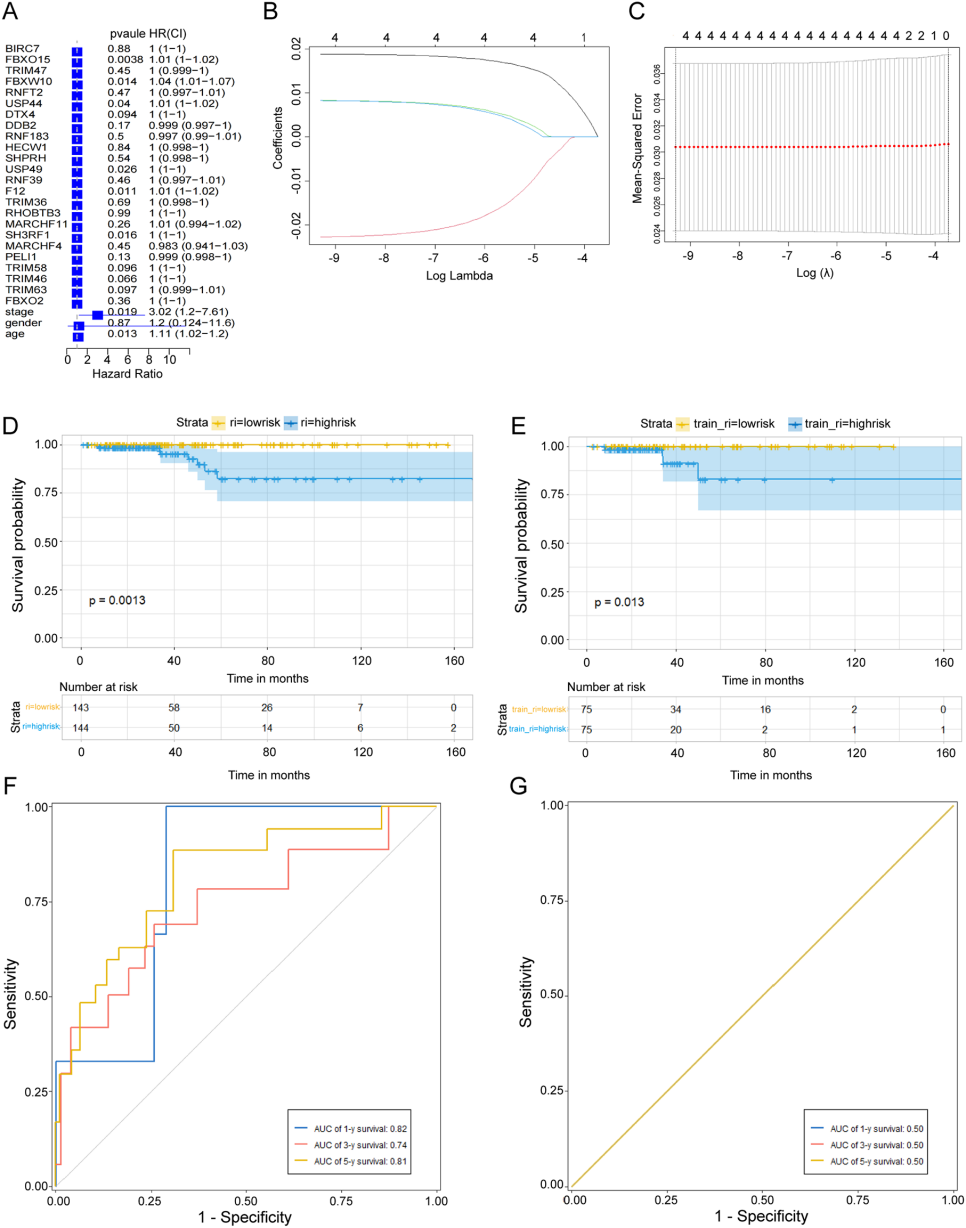
Identification and validation of the ubiquitination-related genes. **(A)** Univariate Cox regression analysis identifying 24 prognostic DEGs. **(B)** Coefficients of the LASSO analysis. **(C)** The ubiquitination-related signature obtained four prognostic genes with a minimum lambda value. **(D, E)** K-M survival analysis showing a significant survival difference between low- and high-risk THCA across the TCGA-all and TCGA-training subsets. **(F, G)** ROC analysis showing the stable prediction ability of the ubiquitination-related signature across TCGA-all and TCGA-training subsets.

**Figure 4:**
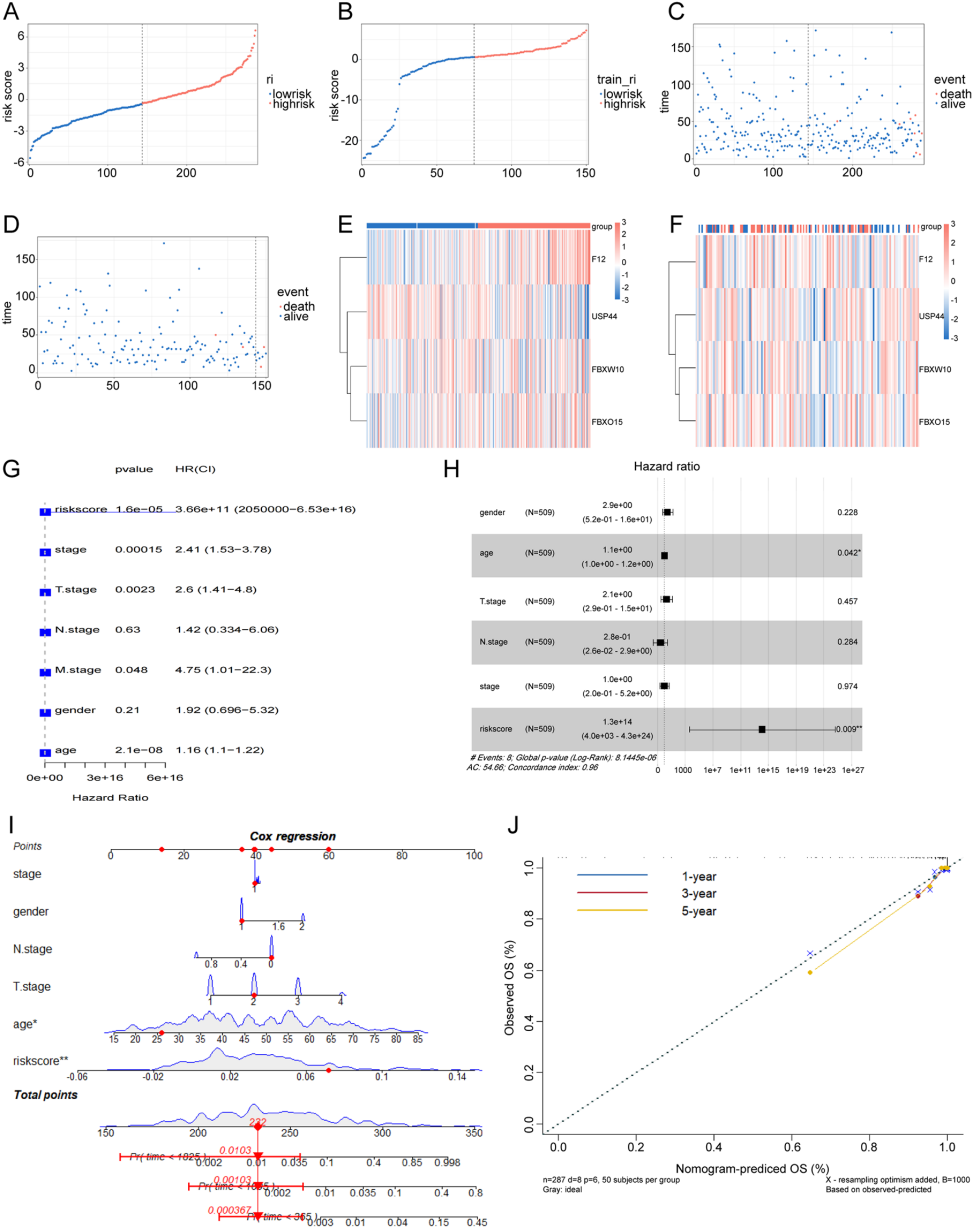
Stability of the ubiquitination-related signature and construction of a nomogram. **(A, B)** Survival curve of the THCA patients across TCGA-all and TCGA-training subsets. **(C, D)** Survival status of the THCA patients across TCGA-all, TCGA-training, and TCGA-test subsets. **(E, F)** Heatmaps showing the expression of signature genes in THCA patients across the TCGA-all, TCGA-training, and TCGA-test subsets. **(G)** Univariate Cox regression analysis of the association between clinicopathological factors (including the risk score) and overall survival of patients in the group of high-risk and low-risk. **(H)** Multivariate Cox regression analysis of the association between clinicopathological factors (including the risk score) and overall survival of patients e in the group of high-risk and low-risk. **(I)** The nomogram was constructed with the ubiquitination-related signature, age, gender, T stage, M stage, N stage, and clinical stage. **(J)** The calibration curve was used to estimate the prediction accuracy of the nomogram.

A univariate Cox regression analysis was used to identify DEGs among tumor and normal tissues. Four risk genes, including F12, USP44, FBXW10, and FBX015 (Hazard Ratio, 1.01, 1.01, 1.04, and 1.01, respectively) (**Figure 3A**). Subsequently, LASSO analysis narrowed down the candidate genes, and 4 ubiquitination-related genes with optimal λ values were screened (**Figures 3B, C**). 4 ubiquitination-related genes were identified and used to construct the risk formula: (0.018805721 * F12 expression) + (-0.022707754 * USP44 expression) + (0.008290455 * FBXW10 expression) + (0.008260817 * FBXO15 expression). Based on the median risk score, patients were divided into low- and high-risk subgroups. A K-M survival analysis was conducted for the training and test subsets, revealing that low-risk THCA had a significantly better prognosis than high-risk THCA (p = 0.0013 and p = 0.013, respectively; **Figures 3D, E**). ROC analysis showed that in the TCGA-all subset, the AUCs of 1-, 3-, and 5-year survival were 0.82, 0.74, and 0.81; in the TCGA-train subset, the 1-, 3-, and 5-year survival AUCs were 0.5, 0.5, and 0.5, revealing the very stable predictive ability of the signature (**Figures 3F, G**). Setting the median risk score as the threshold and plotting the survival status revealed that nearly all patients with high-risk THCA died, thereby further demonstrating the stability of our ubiquitination-related signature (**Figures 4A–D**). Heatmaps showed the expression of four signature genes in low- and high-risk THCA, and the trends were consistent in the train subset (**Figures 4E, F**).

### Construction of a nomogram

Univariate and multivariate Cox regression analyses showed that age and risk score could be independent prognostic indicators (p<0.05) (**Figures 4G, H**). A nomogram was constructed based on sex, T stage, N stage, age, clinical stage, and risk score to build a more useful tool for individuals (**Figure 4I**). The final nomogram scores of each patient obtained by combining all items can be used to predict 1-, 3-, and 5-year survival rates. The calibration curves showed that the nomogram had perfect accuracy in predicting survival (**Figure 4J**). The C-index for this nomogram was 0.763 at 1-year, 0.806 at 3-years, and 0.760 at 5-years, suggesting high accuracy. Moreover, DCA results also indicated a good net benefit (**Supplementary Figure S1**), suggesting that the nomogram is highly practical for clinical application.

### Clinical correlation analysis of ubiquitination-related signature

The relationships between age, sex, T stage, N stage, M stage, and clinical stage, and the signature were calculated to explore the clinical correlation of the signature. The results showed that the risks were higher in T4 than in T1 (p = 0.04, **Supplementary Figure S2**). Although the survival difference between low- and high-risk THCA has been demonstrated in the training and test subsets, subgroup analysis was conducted to confirm the predictive ability of the signature. The results showed a significantly better prognosis in low-risk THCA than in high-risk THCA in the subgroups of male, female, T3–4, and stage III–IV (p = 0.015, p = 0.0077, p = 0.00015, p = 0.0055, respectively; **Supplementary Figure S3**). Moreover, the signature was further validated in two independent datasets. In GSE165724, the risk scores were significantly elevated in primary tumor tissues compared to both adjacent normal tissues (p = 3.6e-07) and distant normal thyroid tissues (p =0.0087) (**Supplementary Figure S4A**). In GSE150899, the risk scores were significantly higher in lymph node metastasis tissues compared to normal thyroid tissues (**Supplementary Figure S4B**) (p = 0.018). This finding suggests that the signature exhibits significant correlations with clinicopathological characteristics and underscores its relevance to tumor development and aggressiveness.

### Comparison of immune infiltration

**Figure 5:**
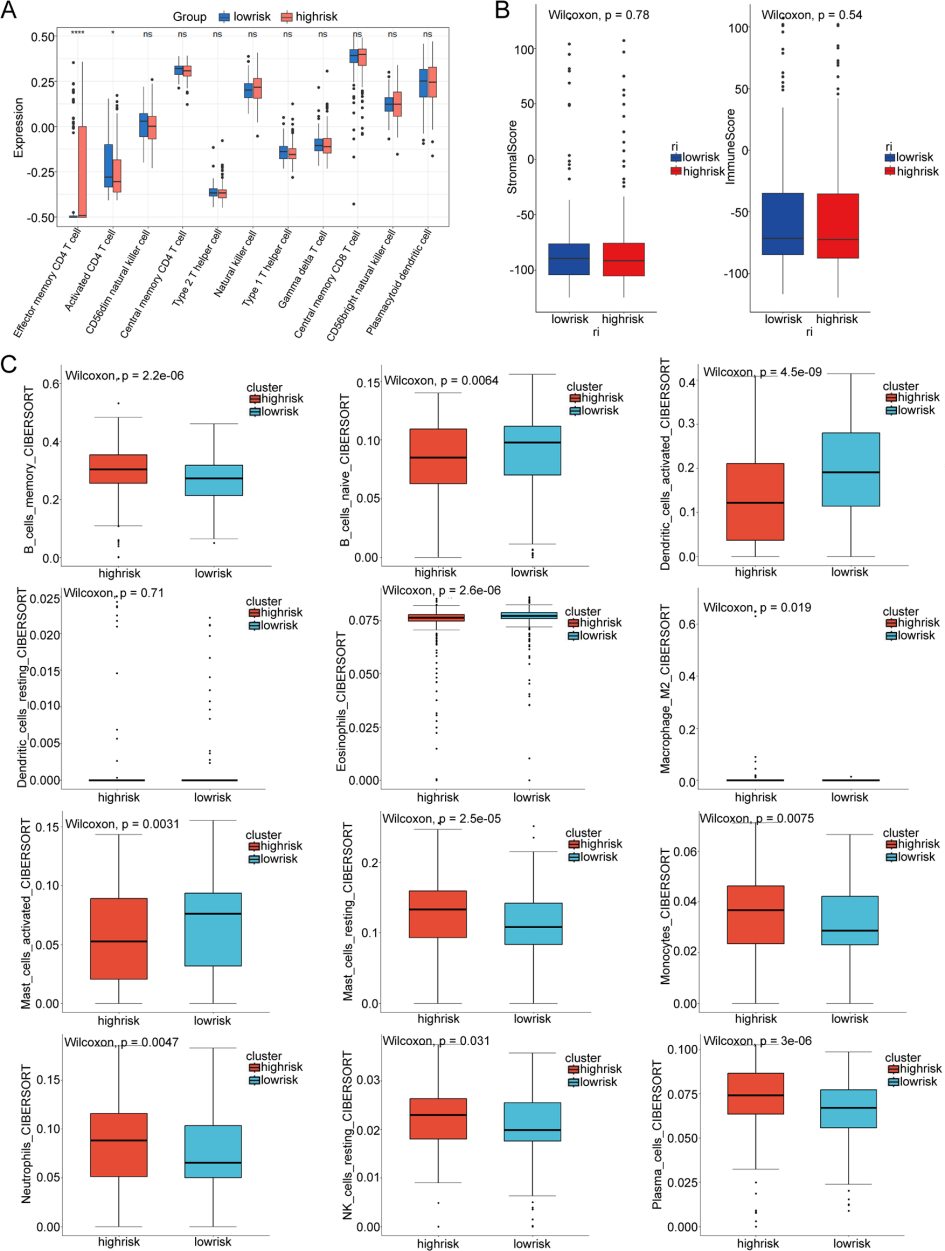
Immune cell infiltration pattern in low- and high-risk THCA. **(A)** Correlation analysis of risk score and diverse immune cells using the ssGSEA algorithms. **(B)** Boxplot showing the expression difference of immune score and stromal score in low- and high-risk THCA. **(C)** Violin plot showing infiltration of CD8 T cells, cytotoxic lymphocytes, endothelial cells, fibroblasts, monocytic lineage, myeloid dendritic cells, neutrophils, NK cells, and T cells in low- and high-risk THCA. ns, no significance. * indicated p <0.05; ** indicated p <0.01; *** indicated p <0.001.

CIBERSORT, ssGSEA, and ESTIMATE were used to understand differences in immunological function. The ssGSEA analysis showed that immunological function was not different between low- and high-risk groups (**Figure 5A**). A lack of difference was observed between the low-risk group and the high-risk group in the immune score and stromal score (**Figure 5B**). Moreover, the high-risk group showed a lower number of immune cells, such as B cells naive and mast cells activated (**Figure 5C**).

### Immunotherapy response

**Figure 6:**
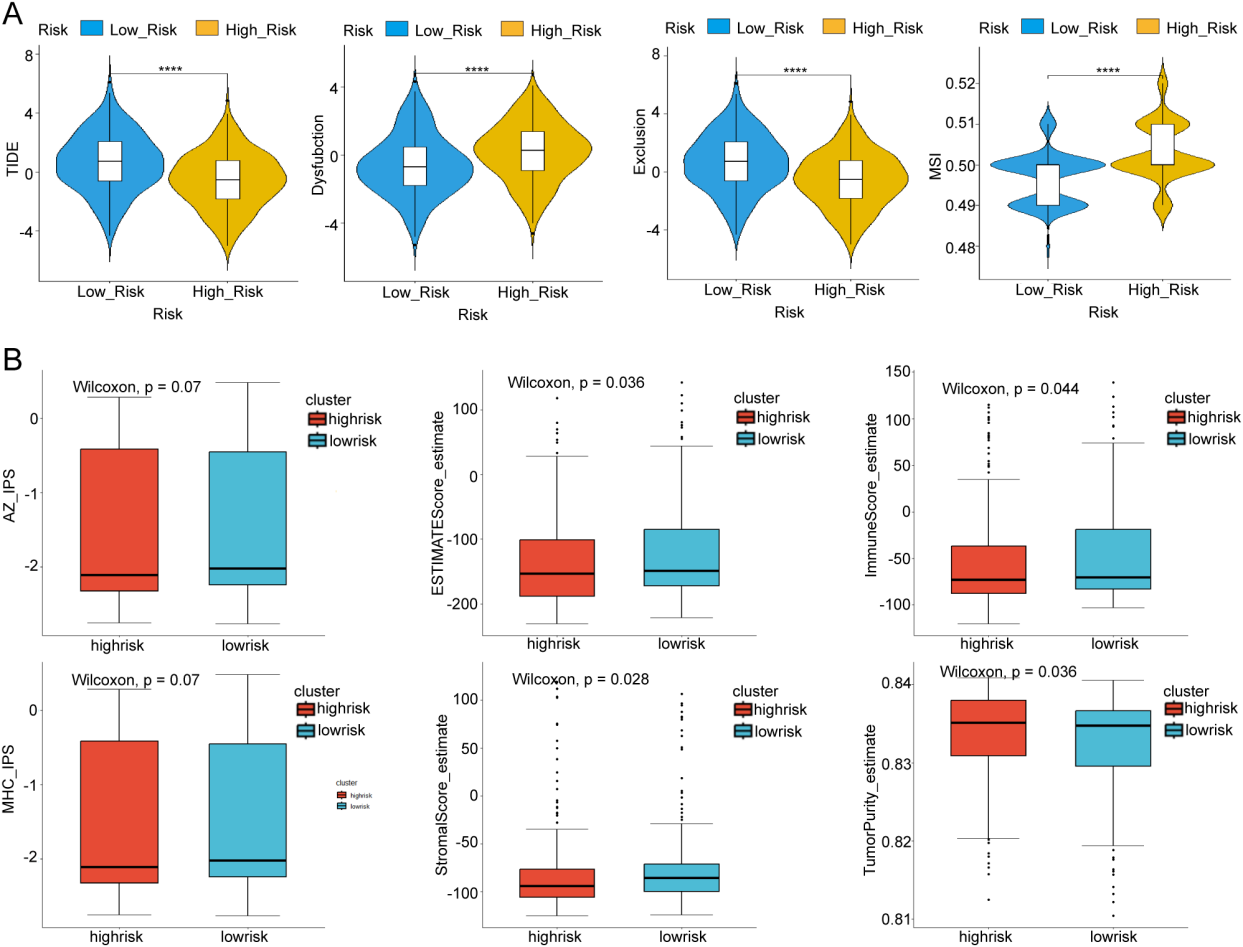
Immunotherapy response of low- and high-risk THCA. **(A)** Difference of the TIDE score between low- and high-risk THCA. **(B)** IPS score of the low- and high-risk THCA in the Estimate score, immune score, stromal score, and Tumour purity subgroups. * indicated p <0.05; ** indicated p <0.01; *** indicated p <0.001.

Dysfunction and MSI were lower in the low-risk subtype, indicating that patients with low-risk THCA may have a better response to immunotherapy (p < 0.05, **Figure 6A**). In addition, the TIDE score and exclusion levels were higher in the low-risk subtype, supporting a better response to low-risk THCA (p < 0.05, **Figure 6A**). IPs in the six subgroups were explored. The results showed that, across the estimated score, immune score, stromal score, and Tumor purity subgroups, low-risk THCA exhibited higher IPS (p = 0.036, p = 0.044, p = 0.0028, and p = 0.036, respectively), which predicted a better immunotherapy response (**Figure 6B**).

### Functional enrichment analysis for low- and high-risk THCA

**Figure 7:**
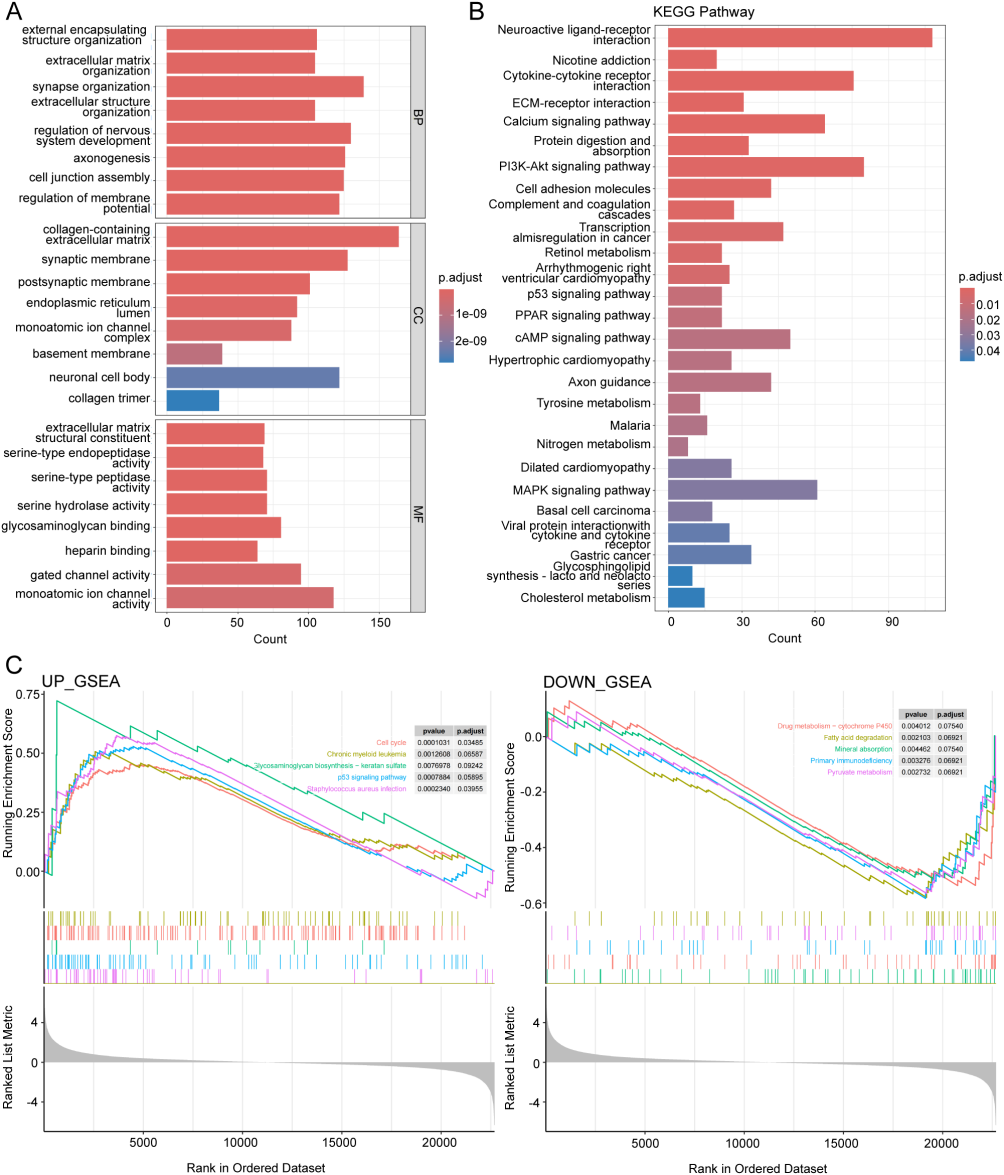
Functional enrichment analysis of low- and high-risk THCA. **(A)** GO enrichment results across TCGA-THCA, including BP, CC, and MF analysis. **(B)** KEGG enrichment results showing the top related pathways across TCGA-THCA. **(C)** GSEA identifying the top five gene sets in low- and high-risk THCA.

To further investigate the putative cellular function and pathway of low- and high-risk THCA, the DEGs between the two subtypes were identified with the criteria of |log2FC|> 2 and p < 0.05. BP analysis showed that the top three enriched functions were external encapsulating structure organization, extracellular matrix organization, and synapse organization (**Figure 7A**). CC analysis revealed that the top three enriched functions were collagen-containing extracellular matrix, synaptic membrane, and postsynaptic membrane (**Figure 7A**). MF analysis confirmed that the extracellular matrix structural constituent, serine-type endopeptidase activity, and serine-type peptidase activity were the most enriched functions (**Figure 7A**). KEGG analysis demonstrated that the top five enriched pathways were neuroactive ligand-receptor interaction, nicotine addiction, cytokine-cytokine receptor interaction, ECM-receptor interaction, and calcium signaling pathway (**Figure 7B**). GSEA revealed differential molecular functions of the THCA cohort. Cell cycle, chronic myeloid leukemia, glycosaminoglycan biosynthesis -keratan sulphate, p53 signaling pathway, and *Staphylococcus aureus* infection were upregulated (**Figure 7C**). The top five downregulated pathways were drug metabolism -cytochrome P450, fatty acid degradation, mineral absorption, primary immunodeficiency, and pyruvate metabolism (**Figure 7C**).

### Drug sensitivity in low- and high-risk THCA

Drug analysis was conducted for low- and high-risk THCA to predict the sensitivity of several common drugs. Acetalax, palbociclib, sorafenib, and uprosertib showed higher sensitivity in low-risk THCA (p = 2.9e-12, p = 0.038, p = 0.039, and p = 0.0057, respectively). Moreover, in high-risk THCA, bortezomib, carmustine, dabrafenib, entinostat, erlotinib, LY2109761, mitoxantrone, nelarabine, obatoclax mesylate, and sepantronium bromide. Venetoclax and zoledronate were less sensitive than those in high-risk THCA (p = 4.2e-06, p = 0.0034, p = 0.0061, p = 0.011, p = 0.0088, p = 1.1e-07, p = 1.3e-06, p = 0.005, p = 2.22e-16, p = 0.0015, p = 0.0035, respectively; **Supplementary Figure S5**).

### Expression of signature genes in THCA

**Figure 8:**
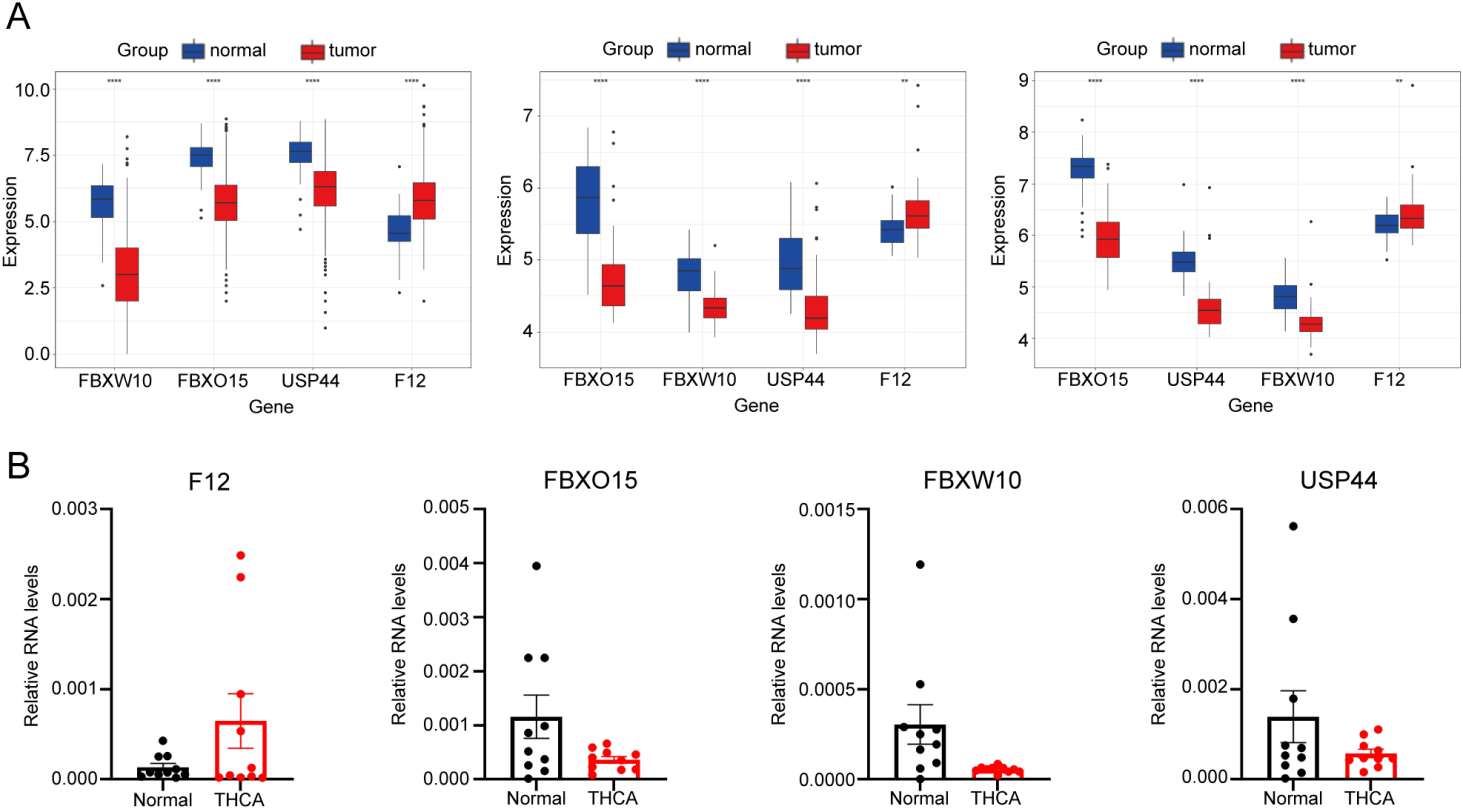
Expression of the signature gene. **(A)** Gene expression differences across the TCGA dataset, GSE60542, and GSE33630. **(B)** RT-qPCR verifying the gene transcription in tumour and normal cells. * indicated p <0.05; ** indicated p <0.01; *** indicated p <0.001.

To further demonstrate the abnormal expression of the four signature genes in THCA, expression analysis was performed using three independent datasets, namely, TCGA-THCA, GSE60542, and GSE33630. USP44, FBXW10, and FBXO15 were more highly expressed in THCA than in normal samples (p < 0.05), whereas the expression of F12 was lower than that in tumor samples (p < 0.05) (**Figure 8A**).

In a real-world experiment, the RT-qPCR results were consistent with the bioinformatic analysis, confirming that F12 was expressed at higher levels in thyroid cancer cells (p < 0.05), while FBXO15, FBXW10, and USP44 were expressed at lower levels (p < 0.05) (**Figure 8B**) (**Supplementary Table S4**).

**Figure 9:**
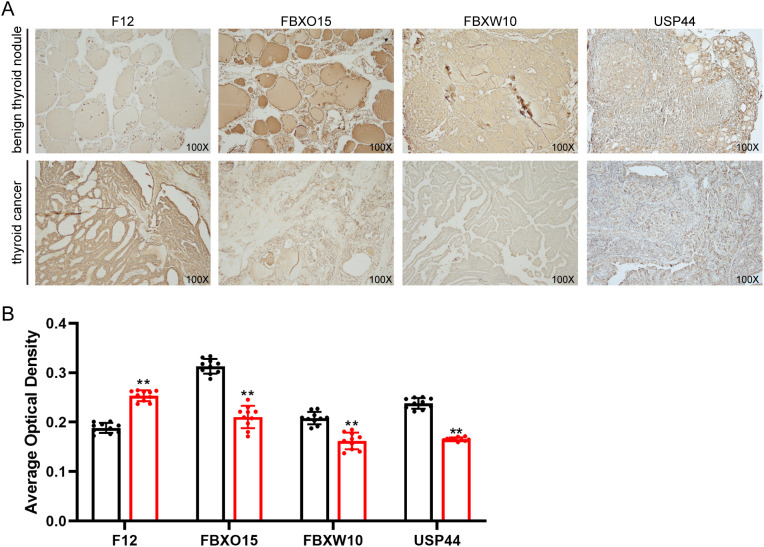
Representative immunohistochemical staining of F12, FBXO15, FBXW10, and USP44 in benign thyroid nodule tissue and thyroid cancer tissue. **(A)** Immunohistochemical image of F12, FBXO15, FBXW10, and USP44 in thyroid cancer tissue. **(B)** The average optical density of IHC staining. ** p <0.01.

Immunohistochemistry results revealed that the staining intensity of F12 was higher in thyroid cancer tissue than in benign thyroid nodule tissue in our specimens. The staining intensity of FBXO15, FBXW10, and USP44 was higher in benign thyroid nodule tissue than in thyroid cancer tissue in our specimens, consistent with bioinformatic analysis (**Figure 9**).

## Discussion

With the development of Precision Medicine, targeted diagnosis and therapy functions interfere with the critical molecules involved in the development of cancer (7). Previous research has shown that the activation of the cGAS-STING pathway triggers antitumor immunity, thereby enhancing IFN-I signaling (48). Golgi transport 1B (GOLT1B) has been associated with most cancer cellular malignant behaviors and immune responses in colorectal and lung cancer, targeting GOLT1B influences the tumor microenvironment, as well as cancer immunotherapy effects (49). In osteosarcoma, the SUMOylation-related gene ZNF451 ZNF451 was found to significantly enhance the growth, migration, and invasion of resistant cells while reducing their sensitivity to cisplatin. ZNF451 also regulates CD8+ T cell function, leading to their exhaustion and transition to the CD8T, thereby altering the tumor immune microenvironment (50).

Protein ubiquitination is involved in various biological processes to regulate the growth or death of tumor cells (51). E3s/DUBs regulate numerous pathways by ubiquitinating substrates or altering ubiquitination levels. More comprehensive research into E3/DUB will help expand therapeutic targets and identify effective biomarkers for cancer. Ubiquitination plays a key role in the THCA development. The USP family members, a crucial subset of DUBs, exhibit divergent roles in THCA. Inactivated USPP44 accelerated the progression of thyroid cancer via the degradation of p21 (52). USP15 could promote THCA via deubiquitinating and stabilizing HMGB1 (53). Therefore, it is worth exploring a prognostic prediction model for patients based on E3s/DUB. Although some signature gene-based prediction models for thyroid carcinoma have been reported (54–56), studies have focused on constructing a model of ubiquitination genes in THCA. A novel signature of ubiquitination-related genes was identified, stably predicting prognosis and immunotherapy response, providing global evidence for ubiquitination in THCA. A subgroup analysis revealed that the ubiquitination-related signature can serve as a biomarker for the prognosis of THCA in patients, females, males, T3-4, and clinical stage III-IV.

The signature comprised four genes: F12, USP44, FBXW10, and FBX015. Coagulation factor XII (F12), also called the Hageman factor, is a single-chain zymogen with a molecular weight of about 80 kDa (57). Low F12 plasma levels were observed in patients with colorectal, gastrointestinal, and lung cancer (58–60). Moreover, F12 could promote ovarian cancer metastasis by transforming monocytes/macrophages into tumor-associated macrophage-like cells (61). Previous study confirmed that F12 expression is higher in papillary thyroid cancer and linked to immune cell infiltration in papillary thyroid cancer (62). Similarly, in this study, F12 was highly expressed in THCA tissues. USP44 is a member of the deubiquitinase family and plays an important role in cell growth. USP44 regulates chromosome separation in anaphase by deubiquitinating cdc20, a cofactor of the APC gene (63, 64). USP44 is upregulated in cancer stem cell (CSC) subpopulations and contributes to aggressive behavior in breast cancer (65). In THCA, USP44 inhibited cell proliferation and induced cell cycle arrest by stabilizing p21 protein (52). FBXW10, also named C17Orf1 protein, C17orf1A protein, HREP, SM25H2, and SM2sH2, is located on 17p12 and is a member of the FBXW subgroup (66). The FBXW10 gene contains more than six exons, spans a length of >17 kb, and encodes a 252 amino acid protein (67). FBXW10 was upregulated in THCA tissues, and its knockdown effectively suppressed THCA cell proliferation (68). FBXO15 has been reported to be a part of the Skp1‐Cullin1‐FBXO15 (SCFFbx15) complex, a ubiquitin E3 ligase (69). FBXO15 plays a key suppressive role in breast cancer by regulating STAT3 and SOX2 ubiquitination and degradation (70). Another study demonstrated that FBXO15 regulates P-glycoprotein/ABCB1 expression through the ubiquitin-proteasome pathway in cancer cells (71). However, the function of FBX015 in patients with THCA remains to be further explored. The association of these genes with several types of cancer has been widely studied. This study confirmed the functions of these genes in THCA.

The TME contains diverse cell types (endothelial cells, macrophages, T cells, dendritic cells, etc.) and extracellular components (extracellular matrix, cytokines, hormones, etc.) surrounding tumor cells, which affect tumor progression (72). The thyroid gland is one of the endocrine organs involved in human immunity. The TME of THCA becomes complex because of the effects of other diseases, such as Hashimoto’s lymphocytic thyroiditis. Previous studies confirmed the coexistence of Hashimoto’s disease and PTC (73). However, some studies have reported that Hashimoto’s thyroiditis may be tumor-protective. Others indicate that it is tumor-promoting; they all demonstrate that the thyroid microenvironment is pivotal to THCA progression (74). In the analysis of immune cell infiltration, M2 macrophages and NK cells showed higher infiltration rates in high-risk THCA; B cells and CD4 T cells presented higher infiltration rates in low-risk THCA. The tumor microenvironment (TME) is the core mediator of tumor development and therapeutic resistance. It contains various types of cells, such as infiltrating immune cells, stem cells, cancer-associated fibroblasts, and vascular cells (75). Understanding the interactions between tumors and their microenvironment is a growing research field. High levels of immune infiltrating cells and immune function scores are often associated with a favorable prognosis (76). In addition, diverse immune cell types were significantly different between the two subtypes, demonstrating that this model functions as a regulator of tumor-infiltrating immune cells in THCA, and that targeting these genes may improve the efficacy of immunotherapy in THCA.

Functional enrichment analysis revealed that various pathways play putative roles in low- and high-risk THCA, such as cytokine-cytokine receptor interaction, extracellular matrix organization, and p53 signaling pathway. Cytokine–cytokine receptor interactions have been reported to be strongly associated with the risk of diverse cancers. The intricate complexity of cancer becomes evident upon microscopic examination of solid tumors. The tumor microenvironment (TME) is a highly structured ecosystem containing cancer cells surrounded by diverse non-malignant cell types, and they are collectively embedded in an altered, vascularized extracellular matrix (77). TME cells and their secreted molecules play critical roles in the pathogenesis of cancer and thus represent attractive therapeutic targets (78). The infiltration of different immune cells in low- and high-risk THCA may partly account for the differences in malignancy and prognosis. P53 is mutated in more than 50% of human cancers, and the remaining of 100% involve biological inactivation of its pathway, including MDM2 amplification, loss of p14ARF, and mutations in activating kinases, such as ATM and Chk2 (79). The loss of p53 pathway function gives cancer cells a survival advantage, allowing them to bypass resolution of oncogenic signals and DNA damage and continue abnormal proliferation (80). Our analysis further demonstrated that ubiquitination-related signatures are associated with the p53 signaling pathway.

Bioinformatics analysis investigated the predictive value of ubiquitination-related genes for thyroid cancer prognosis, immunotherapy response, and drug sensitivity, and discussed the putative mechanisms of these genes. Regarding limitations, the drug-sensitivity predictions are derived from computational analyses and have not been experimentally validated. Future work should include *in vitro* and *in vivo* studies to functionally confirm these predictions. Next, the sample size for the RT-qPCR and IHC validation is relatively small. However, it is important to note that the results from these samples were highly consistent and statistically significant, providing a robust proof of concept. This experimental evidence strongly supports the bioinformatic findings and confirms the feasibility of our approach. While further validation in larger, independent patient cohorts is necessary to fully assess the clinical relevance and generalizability of the signature, the compelling concordance observed in this initial cohort lays a solid foundation for such future studies. In conclusion, this study identified a novel ubiquitination-related prognostic signature containing four genes. The four-gene signature has better economic viability compared with other clinical gene predictive models, such as the 21-gene recurrence score and 70-gene signature test (MammaPrint) for breast cancer (81, 82). The risk score derived from the signature can independently predict survival and immunotherapy benefit in patients with THCA. Our new ubiquitination-related model may be used for THCA-targeted therapy in the future.

## Data Availability

The original contributions presented in the study are included in the article/**Supplementary Material**. Further inquiries can be directed to the corresponding authors.

## Supplementary material

Supplementary Figure 1 DCA for assessment of the clinical utility of the nomogram in 1, 3, and 5 years.

Supplementary Figure 2 Correlation analysis showing that the ubiquitination-related signature is associated with age, gender, stage, T stage, M stage, and N stage.

Supplementary Figure 3 K-M survival analysis presenting the significance of prognosis between low- and high-risk THCA in subgroups of age > 65, female, male, N0, N1, T1-2, T3-4, stage I-II, and stage III-IV.

Supplementary Figure 4 Validation of the TCGA-derived Prognostic Signature in Independent Datasets. **(A)** In dataset GSE165724, the risk score was highest in PTC tumor tissues, compared to normal thyroid tissues and tumor-adjacent thyroid tissues. **(B)** In dataset GSE150899, the risk score is elevated in lymph node metastases compared to normal thyroid tissues.

Supplementary Figure 5 Drug sensitivity in low- and high-risk THCA, including Acetalax, Palbociclib, Sorafenib, Uprosertib, Bortezomib, Carmustine, Dabrafenib, Entinostat, Erlotinib, LY2109761, Mitoxantrone, Nelarabine, Obatoclax Mesylate, Sepantronium bromide, Venetoclax, and Zoledronate.
