# Supplementary figures and images for: Identify and validate a novel ubiquitination-related biomarker for thyroid cancer prognosis and immunotherapy
Source: Front Oncol. 2026 Jan 16;15:1542784. doi: 10.3389/fonc.2025.1542784 (PMC12855127; doi:10.3389/fonc.2025.1542784)

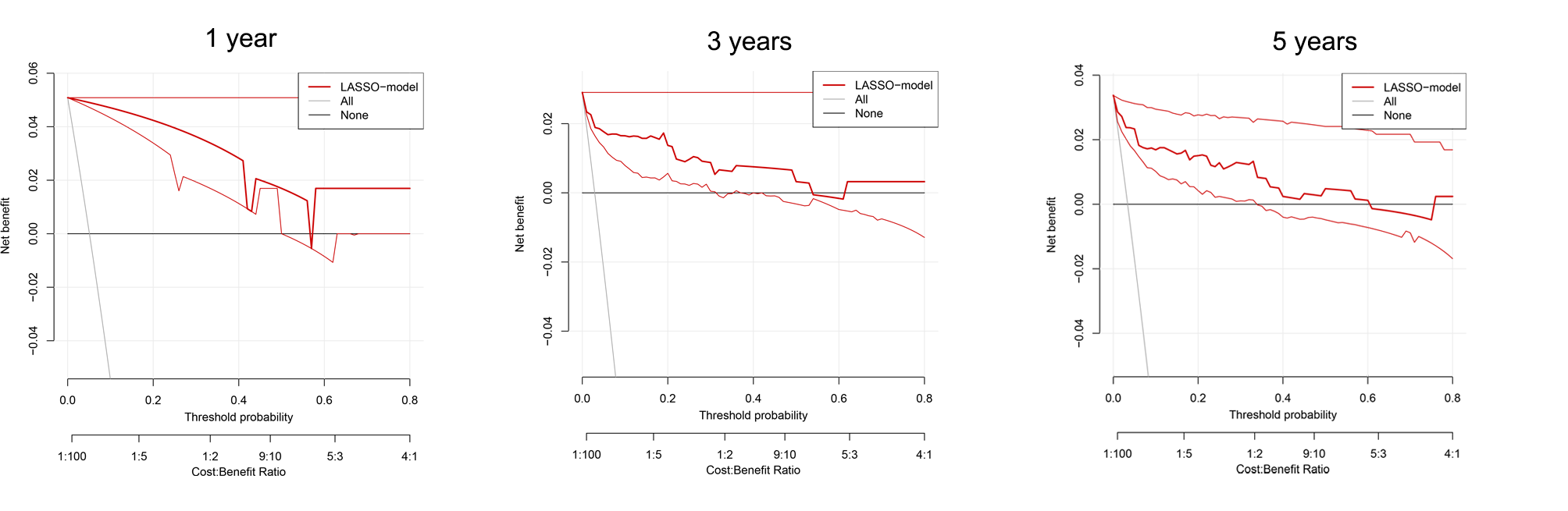

Supplement: Supplementary Figure 1 — DCA for assessment of the clinical utility of the nomogram in 1, 3, and 5 years. [file Image1.tif]

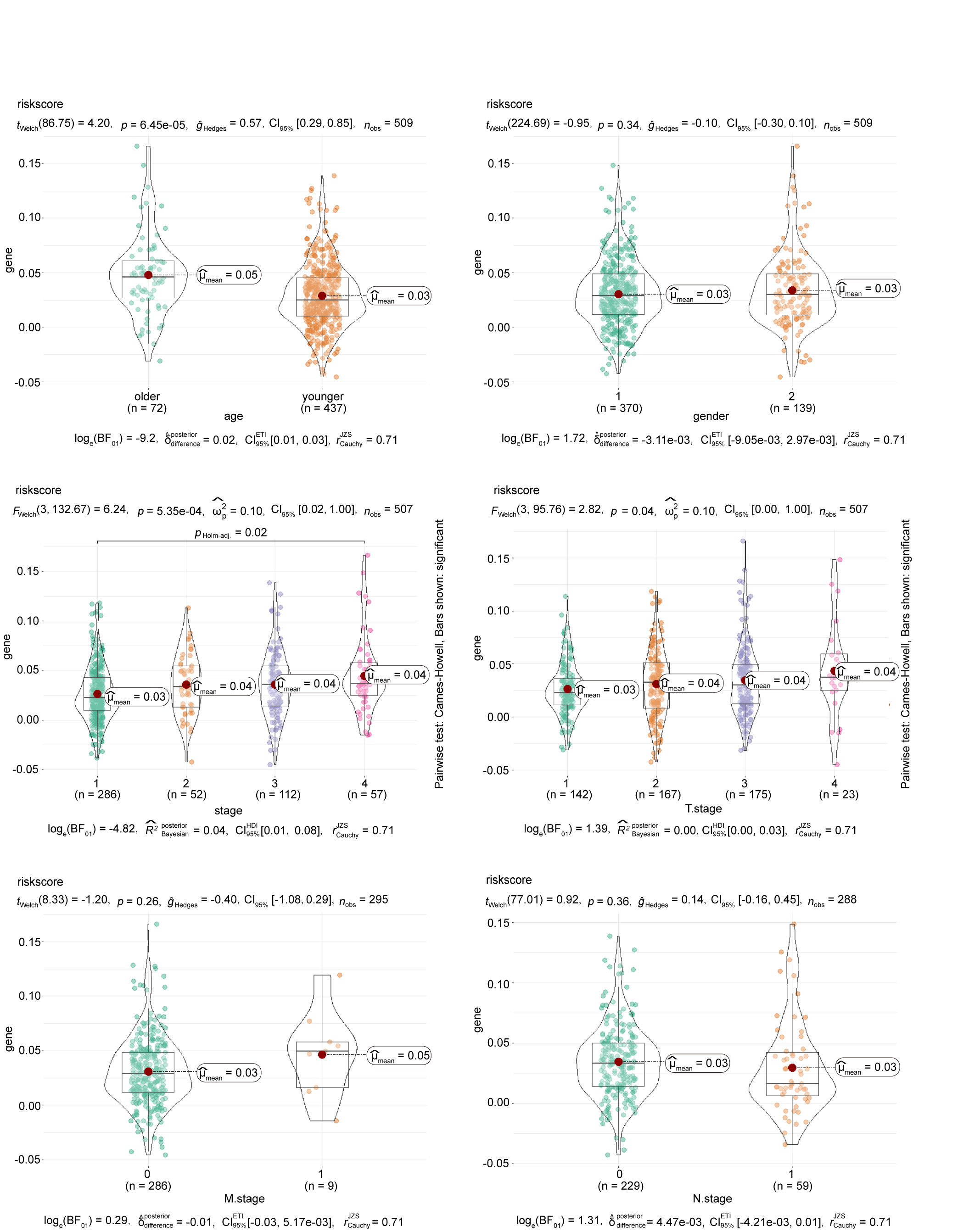

Supplement: Supplementary Figure 2 — Correlation analysis showing that the ubiquitination-related signature is associated with age, gender, stage, T stage, M stage, and N stage. [file Image2.tif]

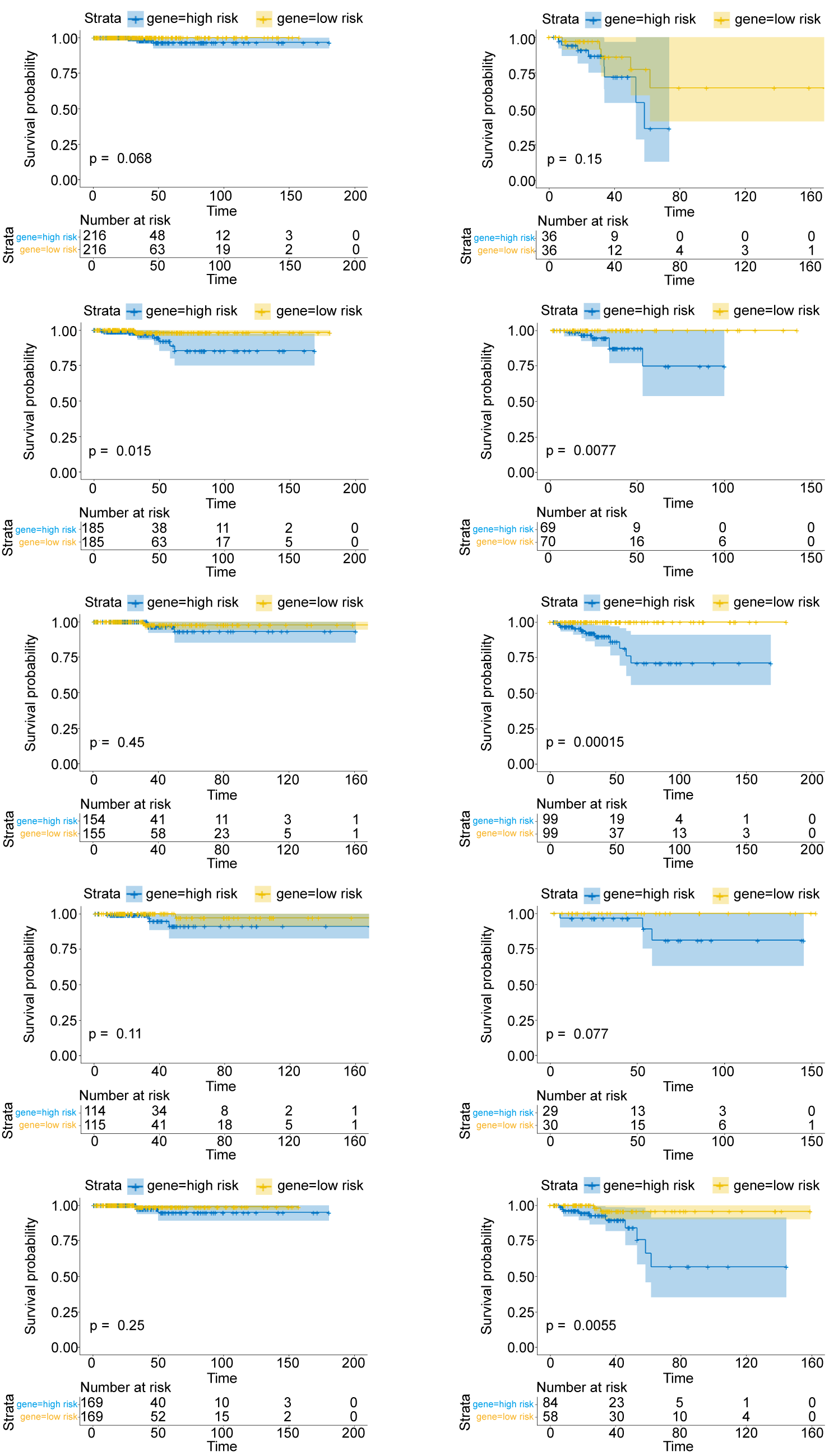

Supplement: Supplementary Figure 3 — K-M survival analysis presenting the significance of prognosis between low- and high-risk THCA in subgroups of age > 65, female, male, N0, N1, T1-2, T3-4, stage I-II, and stage III-IV. [file Image3.tif]

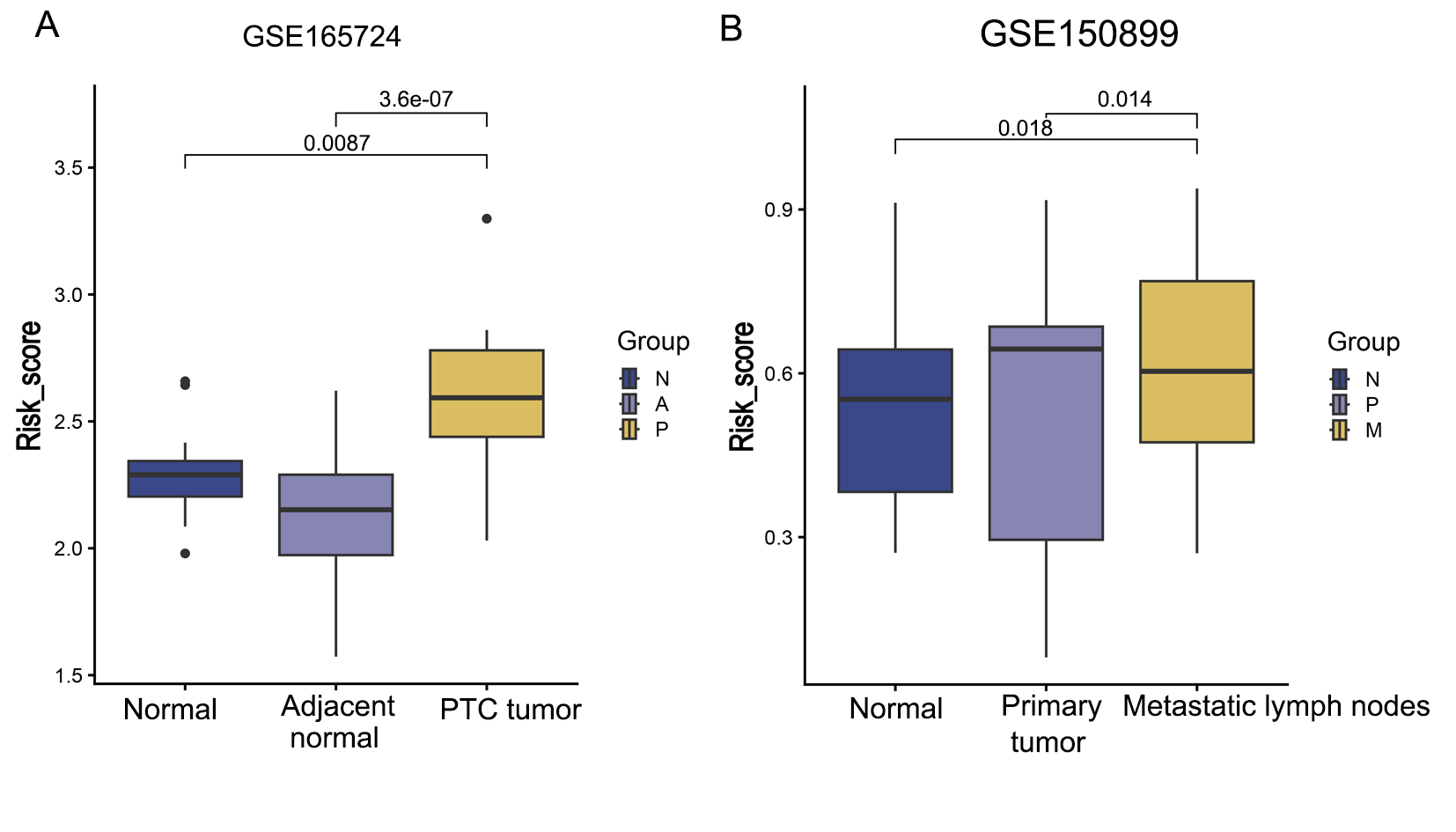

Supplement: Supplementary Figure 4 — Validation of the TCGA-derived Prognostic Signature in Independent Datasets. (A) In dataset GSE165724, the risk score was highest in PTC tumor tissues, compared to normal thyroid tissues and tumor-adjacent thyroid tissues. (B) In dataset GSE150899, the risk score is elevated in lymph node metastases compared to normal thyroid tissues. [file Image4.tif]

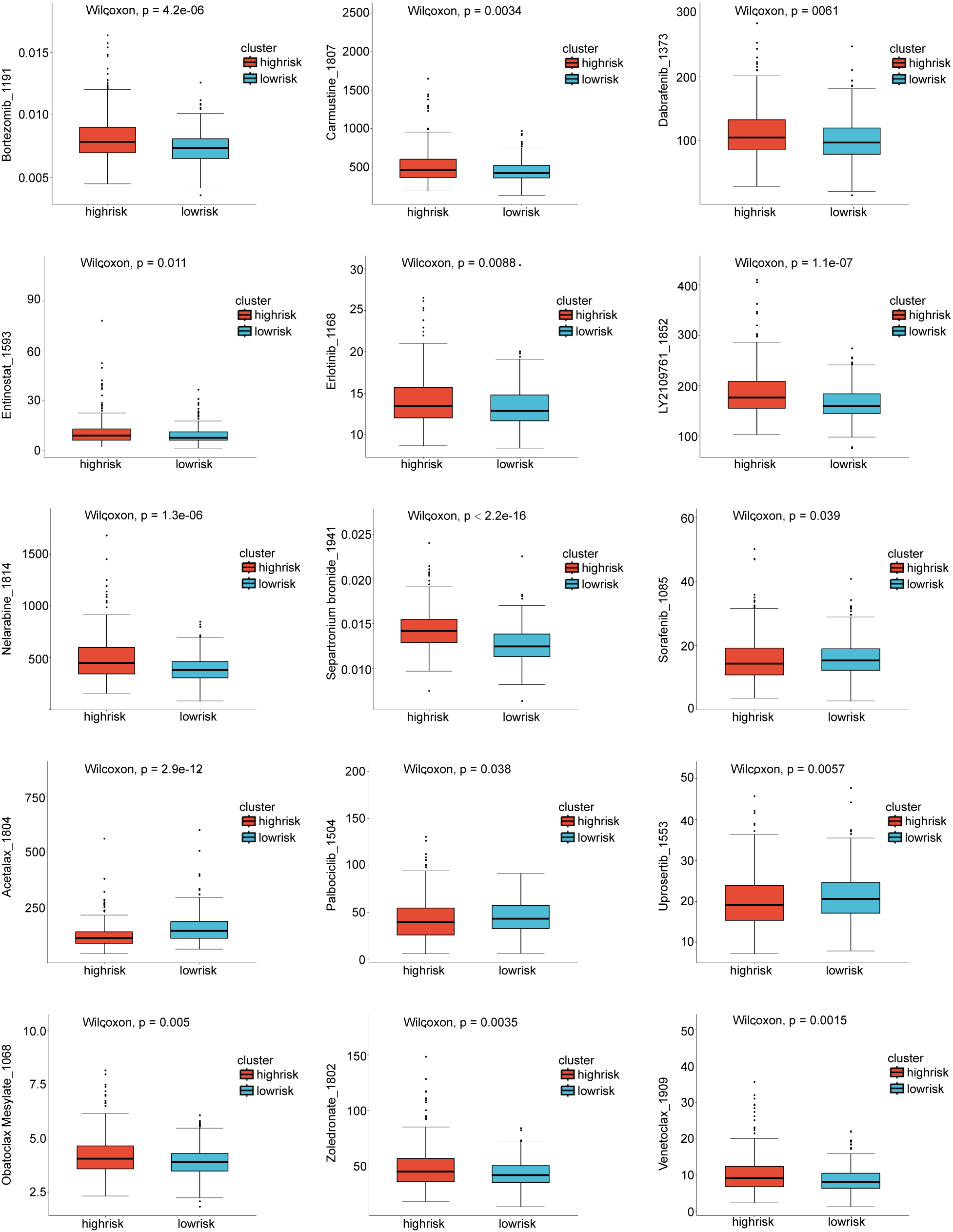

Supplement: Supplementary Figure 5 — Drug sensitivity in low- and high-risk THCA, including Acetalax, Palbociclib, Sorafenib, Uprosertib, Bortezomib, Carmustine, Dabrafenib, Entinostat, Erlotinib, LY2109761, Mitoxantrone, Nelarabine, Obatoclax Mesylate, Sepantronium bromide, Venetoclax, and Zoledronate. [file Image5.tif]
